# Case report: Onychopapilloma in a patient with *BAP1* tumor predisposition syndrome—a useful clinical marker?

**DOI:** 10.1007/s10689-026-00558-z

**Published:** 2026-04-22

**Authors:** Emilie Sjøstrøm, Lise Barlebo Ahlborn, Elisabeth Victoria Eliesen, Karin Wadt, Ulrikke Lei, Anna Byrjalsen

**Affiliations:** 1 Department of Clinical Genetics, Rigshospitalet, Copenhagen University Hospital, Copenhagen, Denmark; 2 Department of Genomic Medicine, Rigshospitalet, Copenhagen University Hospital, Copenhagen, Denmark; 3 Department of Clinical Medicine, Copenhagen University, Copenhagen, Denmark; 4 Department of Dermatology and Allergology, Herlev and Gentofte University Hospital, Copenhagen, Denmark

**Keywords:** BAP1, Onychopapilloma, Mesothelioma, Cancer predisposition, Germline

## Abstract

The *BAP1* gene encodes a tumor suppressor protein implicated in *BAP1* tumor predisposition syndrome (*BAP1*-TPDS), a hereditary condition associated with an increased risk of uveal and skin melanoma, mesothelioma, and renal cancer. In this case report, we describe a male patient diagnosed with mesothelioma carrying a germline *BAP1* variant. The patient exhibited severe nail abnormalities affecting all ten fingernails and several toenails. To our knowledge, the association between onychopapilloma and pathogenic germline *BAP1* variants has only been reported once in the literature. The patient had experienced nail abnormalities since adolescence. Based on these findings, we propose that polydactylous onychopapilloma may serve as a clinical marker of *BAP1*-TPDS in otherwise asymptomatic individuals, and could potentially aid in early identification of at-risk carriers.

## Introduction

The *BAP1* gene and its resulting protein (BRCA1-associated protein (1) is a tumor suppressor in humans [1]. *BAP1* tumor predisposition syndrome (*BAP1*-TPDS) is caused by germline pathogenic variants in *BAP1* and is inherited in an autosomal dominant manner. *BAP1*-TPDS is associated with a high risk of uveal malignant melanoma (UMM), cutaneous malignant melanoma (CMM), malignant mesothelioma (MMe) [2–5], renal cell carcinoma (RCC) [6], and to a lesser degree basal cell carcinoma (BCC) [7], meningioma and cholangiocarcinoma [8]. Besides the increased cancer risk, *BAP1-*TPDS has been associated with benign melanocytic tumors known as *BAP1*-inactivated melanocytomas (BIMs) [5]. These resemble common intradermal nevi, and may therefore easily be overlooked. However, a publication from Lebensohn et al. found benign nail abnormalities, including onychopapilloma in 41/47 patients with *BAP1*-TPDS, but whether this is a definitive marker for the syndrome remains unknown [9].

In this case-report we present a patient with mesothelioma and *BAP1-*TPDS who ‒ on examination ‒ has severe onychopapilloma in all 10 fingernails and several toenails.

## Case description

A 63 year-old male was referred by his general practitioner to the Department of Respiratory Medicine due to complaints of cough, right-sided thoracic pain, and hemoptysis, resulting in the discovery of a right-sided infiltrate and pleural effusion. Until then, the patient had been generally healthy, apart from a history of hypertension.

The patient’s father was in the family known to have had a stomach cancer and passed away at age 37. The paternal grandfather also died of an unknown cancer.

PET-CT revealed a 10.2 cm tumor in the right upper lung lobe with thoracic wall and mediastinal invasion, metastases to the pleura, pericardium, and lymph nodes. Biopsy confirmed epithelioid mesothelioma.

The patient was further referred to the oncology department, where neoadjuvant chemotherapy with cisplatin and pemetrexed was initiated to reduce tumor burden with the initial aim of achieving operability.

Simultaneously, the patient was referred to the department of occupational medicine for evaluation of potential asbestos exposure. This was deemed likely due to the patient’s work as an electrician. No known asbestos exposure was reported in private settings.

Despite three years of oncological treatment covering 15 cycles of different chemotherapies, a control-CT-scan showed progressive disease, and the patient was referred to the Phase 1 clinical trial unit through the oncology department, where patients are offered experimental treatment or treatment within a clinical trial set-up. A genomic tumor profiling was conducted ahead of potential participation in a clinical trial. The tumor profiling covers RNA-sequencing and whole genome sequencing of both tumor and germline.

The analysis identified a germline heterozygote nonsense variant in *BAP1*, c.178C > T, p.(Arg60Ter), VAF (67%). The variant was not previously reported in population databases (gnomAD version 4.1), had so far been reported seven times in ClinVar (Variation ID: 580,226), and co-segregates in families with *BAP1*-related disease [7]. The variant has not been investigated in functional studies and based on ACMG/AMP guidelines the variant was classified as disease causing (Class 5, pathogenic based on fulfillment of PVS1, PM2_supporting, and PP1).

**Fig. 1:**
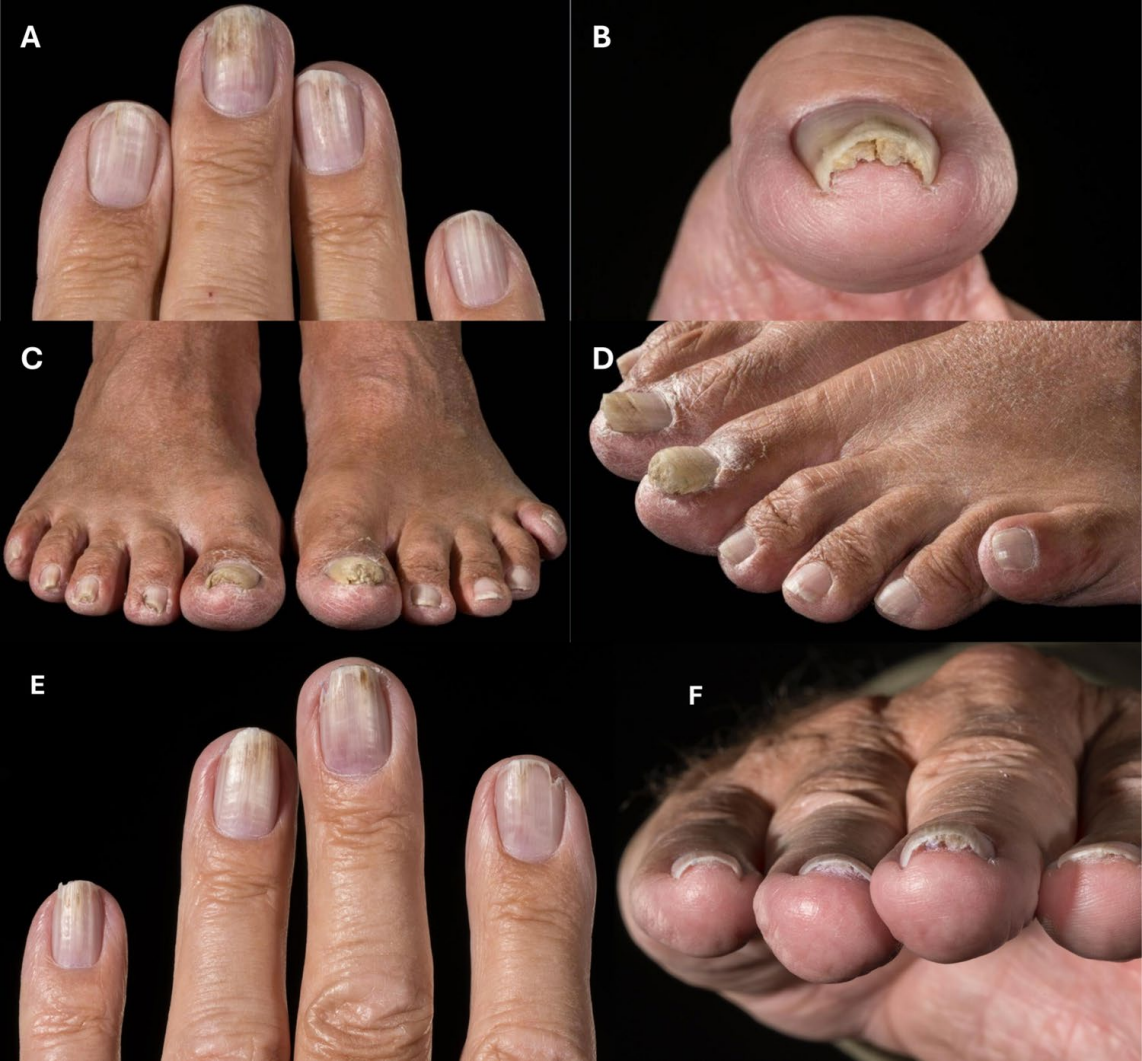
Nail abnormalities in a patient carrying a germline BAP1-variant. **A**: Longitudinal leukonychia with involvement of multiple digits. **B**: Nail of first digit showing subungual hyperkeratosis. **C**: Subungual alterations affecting several digits. **D**: Both halluces with pronounced subungual hyperkeratosis extending proximally. Note that toes 3, 4, and 5 on the left foot appear largely unaffected. **E**: 2–5 digit of the left hand, showing distal longitudinal red and white bands, distal discoloration, distal v-shaped fissuring and a few splinter hemorrhages. **F**: 2–5 digit of the right hand, showing hypercurvature of the nails and prominent subungual hyperkeratosis

Subsequently, the patient was referred for genetic counselling. He arrived with both children (adult son and daughter), who both proved positive for the germline variant. Unprompted the patient asked whether his abnormal nails (Fig. 1) could be associated with *BAP1*-TPDS. It turned out that there had been one previous description of nail onychopapilloma in the literature, and the patient was referred for assessment at the dermatology department. The patient’s son had nail dystrophy in a single fingernail but nowhere else, the daughter had no nail abnormalities.

Assessment at the dermatology department showed various degrees of involvement of all fingernails and several toenails. Of the toenails the first digits were more severely involved. The changes were more severe on fingernails compared to toenails. We found distal longitudinal red and white bands, distal discoloration, distal v-shaped fissuring and a few splinter hemorrhages. We also found hypercurvature of the nail and specifically we found prominent subungual hyperkeratosis. These changes are consistent with a clinical diagnosis of onychopapilloma, a benign neoplasm of the distal nail matrix and the nail bed. The patient reported fingernail changes since his teenage years. Involvement of the toenails was evident approximately 10 years later. He did not describe strenuous manual labor, nor had he issues with pain and described normal growth velocity. He had intermittent infection in one index fingernail after nail clipping. Despite undergoing multiple examinations for fungal infections, all tests returned negative results.

## Discussion

Over the past 15 years, our understanding of *BAP1*-TPDS and its phenotypic spectrum has significantly expanded. In this case report, we build upon the association identified by Lebensohn et al. [9] between nail abnormalities and adult carriers of this syndrome.

Skin cancer associated with *BAP1*-TPDS (melanoma and basal cell carcinoma) are relatively frequent in the general population, and a diagnosis of malignancies may not immediately prompt further evaluation for an underlaying cancer syndrome. In 2011, Wiesner et al. [5] described BAP1-inactivated melanocytomas (BIMs), a benign melanocytic neoplasm, as a key marker for identifying carriers of pathogenic *BAP1* variants. However, BIMs can easily be overlooked due to their clinically unremarkable appearance.

In the study by Lebensohn et al. [9], onychopapilloma was identified in 39/47 patients (83%) with *BAP1*-TPDS, with polydactylous involvement observed in 38/39 patients (97.4%). In contrast, onychopapilloma are uncommon in the general population, and involvement of multiple nails is even less so. The timeframe between the onset of nail changes and the recognition of a germline *BAP1*-variant remains uncertain, as these nail alterations are typically slowly progressive and asymptomatic [9].

Early identification of patients with *BAP1*-TPDS is crucial, as these carriers are at an increased risk of developing multiple malignant neoplasms, necessitating long-term surveillance [10]. It is compelling to consider the potential impact on treatment outcomes and overall survival if a pathogenic germline *BAP1*-variant were detected at an earlier stage. Based on Lebensohn et al. and our findings we would urge further enquiry into family history of cancers and dermatologic investigation in patients presenting with unexplained nail abnormalities affecting multiple nails, particularly when other diagnostic tests have been negative. If there is involvement of multiple nails it could be a cutaneous marker of *BAP1-*TPDS and referral for genetic counseling should be considered.

Conclusively, this case underscores the need for increased awareness of subtle dermatological signs that may serve as early indicators of *BAP1*-TPDS in otherwise asymptomatic carriers. Distribution of knowledge is key to potentially diagnose *BAP1*-TPDS prior to a *BAP1*-associated tumor.

## Data Availability

No datasets were generated or analysed during the current study.

## Declarations

### Informed constent

The authors obtained written consent from the patient for his photographs and medical information to be published in print and online and with the understanding that this information may be publicly available. Patient consent forms were not provided to the journal but are retained by the authors.
